# Comprehensive transcriptome assessment in PBMCs of post-COVID patients at a median follow-up of 28 months after a mild COVID infection reveals upregulation of JAK/STAT signaling and a prolonged immune response

**DOI:** 10.3389/fimmu.2025.1589589

**Published:** 2025-05-30

**Authors:** Serena Fineschi, Joakim Klar, Juan Ramon Lopez Egido, Jens Schuster, Jonas Bergquist, René Kaden, Niklas Dahl

**Affiliations:** ^1^ Department of Public Health and Caring Sciences, Faculty of Medicine, Uppsala University, Uppsala, Sweden; ^2^ Östhammar Health Care Centre, Östhammar, Sweden; ^3^ Science for Life Laboratory, Department of Immunology, Genetics and Pathology, Uppsala University, Uppsala, Sweden; ^4^ Analytical Chemistry and Neurochemistry, Department of Chemistry, Biomedicinskt Centrum (BMC), Uppsala University, Uppsala, Sweden; ^5^ The ME/CFS Collaborative Research Centre, Uppsala University, Uppsala, Sweden; ^6^ Department of Medical Sciences, Uppsala University, Akademiska Sjukhuset, Uppsala, Sweden; ^7^ Clinical Genomics Uppsala, Science for Life Laboratory, Uppsala University, Uppsala, Sweden

**Keywords:** post-COVID, SARS-CoV-2, RNA sequencing, JAK/STAT signaling, interferon signature, ubiquitination, interleukin 9, mitochondrial dysfunction

## Abstract

**Background:**

Post-acute sequelae of SARS-CoV-2 infection (PASC), also known as post-COVID-19 condition (here abbreviated as post-COVID) is an escalating global health issue. The aim of our study was to investigate the mechanisms and clinical manifestations of post-COVID following a mild SARS-CoV-2 infection.

**Methods:**

We analyzed the gene expression profile in PBMCs from 60 middle-aged post-COVID patients and 50 age-matched controls at a median time of 28 months following a mild SARS-CoV-2 infection. The clinical assessments included intensity of post-COVID symptoms, physical and mental fatigue, depression and anxiety. Sixty-seven participants performed a mild exertion ergometer test with assessment of lactate concentrations. Transcriptome analysis was performed on mRNA selected by poly-A enrichment and SARS-CoV-2 RNA fragments were analyzed using the ARTIC protocol.

**Results:**

We identified 463 differentially expressed transcripts in PBMCs, of which 324 were upregulated and 129 downregulated in post-COVID patients. Upregulated genes in post-COVID individuals were enriched for processes involving JAK-STAT signaling, negative regulation of ubiquitination, IL9 signaling, and negative regulation of viral process, suggesting chronic inflammation. Downregulated genes were enriched for processes involving mitochondrial ATP synthesis, and oxidative phosphorylation, suggesting mitochondrial dysfunction. No SARS-CoV-2 gene fragments were detected in PBMCs of patients with post-COVID and no IFN genes were found differentially expressed in post-COVID patients. Post-COVID was associated with elevated lactate levels in blood, both at rest and after a short recovery phase following exertion, suggesting increased anaerobic activity in skeletal muscles. We did not find differences in the transcriptional profiles or clinical manifestations when comparing patients who contracted the infection from early SARS-CoV-2 variants with those who contracted the infection during the period when the Omicron variant was prevalent.

**Conclusions:**

Our findings highlight molecular changes compatible with a persistent immune response in PBMCs of post-COVID subjects at a median follow-up of 28 months after a mild infection, supporting the hypothesis that post-COVID is a chronic inflammatory condition. The upregulation of JAK/STAT signaling suggests a potential therapeutic target in post-COVID.

## Introduction

Post-acute sequelae of SARS-CoV-2 infection (PASC), also known as post-COVID-19 condition (here abbreviated as post-COVID), is defined as the continuation or development of new symptoms 3 months after a probable or confirmed SARS-CoV-2 infection, with these symptoms lasting for at least 2 months in absence of an alternative diagnosis, according to World Health Organisation definition, Delphi consensus, 2021 (1).

Post-COVID occurs in approximately 10% of individuals who experienced a SARS-CoV-2 infection and without correlation to severity of infection (2). The symptoms are variable and may persist for several years (3). Older age, female sex, high body mass index (BMI), smoking, diabetes, and a previous episode of severe COVID-19 are associated with an increased risk for post-COVID (4). With the transition from the pandemic to the endemic phase of COVID-19, the major challenges have now gradually changed from complications associated with the acute infection to the long-term debilitating manifestations associated with post-COVID. The number of post-COVID cases may increase in the future due to the cumulative increased risk with reinfections, particularly in women (5). The effect of vaccination to prevent post-COVID is debated (6) but recent data indicate that vaccinated individuals have a decreased risk for post-COVID manifestations (7).

The increasing global burden of patients with post-COVID now calls for efforts to understand the underlying pathophysiology and for the development of efficient therapies. Several reports have proposed pathophysiological and molecular mechanisms behind the progression from the acute phase of infection to post-COVID. These mechanisms include reactivation of latent virus such as Epstein-Barr virus (EBV) (8), persistence of SARS-CoV-2 antigen in reservoirs (9–11), and increased or persistent systemic inflammation and autoimmunity after clearance of the virus (12).

Transcriptome analysis has been applied in a large number of studies on post-COVID in attempts to clarify the pathophysiology behind the condition and to identify candidate targets for medical intervention (13). For example, analysis of peripheral blood mononuclear cells (PBMCs) in convalescent COVID-19 patients several months after the acute infection has shown perturbed expression of several proinflammatory genes, including the transcription factor NF-kB (14) and members of the Janus kinase/signal transducers and activators of transcription (JAK/STAT) signaling pathway (15).

Furthermore, upregulation of genes important for interferon (IFN) signaling has been reported in adolescents and young women affected by post-COVID in a 6 months follow-up after infection (16). Other studies have shown a reduced or normal IFN signaling in post-COVID (17–19). The reason for the different results is not clear but one explanation may be different follow-up periods from onset of infection to sampling for analysis. Moreover, a 6 months follow-up study on 69 post-COVID patients who recovered from mild, moderate, severe or critical COVID-19 uncovered dysregulation of genes related to transcription, translation and ribosome biosynthesis when compared to 14 non-infected healthy control subjects (18). Interestingly, the same study presented evidence for a dysregulated expression of genes involved in oxidative phosphorylation. Altered mitochondrial function has independently been suggested as a potential mechanism underlying some of the manifestations in post-COVID, such as physical and mental fatigue (20, 21). Lactic acid is a by-product of anaerobic metabolism in the muscles and elevated lactate levels have been suggested as a potential explanation for the post-exertional malaise that accompanies myalgic encephalomyelitis/chronic fatigue syndrome (ME/CFS) and post-COVID. Lactate can easily be quantified in blood and lactate measurements have been proposed as a promising tool in the rehabilitation of both disorders (22). Further indirect evidence supporting the long-term impact of SARS-CoV-2 infection on mitochondrial function comes from a study of twenty-seven patients with post-COVID and sixteen controls, conducted eight months post-infection. This study showed that post-COVID was associated with signs of systemic inflammation and immune dysregulation in T cells, accompanied by impaired regulation of genes involved in heme synthesis (23). Since part of heme synthesis occurs within mitochondria, disrupted mitochondrial function may hypothetically interfere with the regulation of the heme synthesis pathway (24).

It is still unclear if post-COVID is associated with transcriptional changes for years after the acute infection, as most current studies have shorter follow-up periods. Furthermore, it is uncertain if the transcriptional changes associated with post-COVID exist prior to infection, if they appear in the initial phase of infection, or if they develop later during the course of the illness. Notably, a longitudinal study in which blood samples were analysed during the acute phase of COVID-19 and at a one-year follow-up showed that molecular changes associated with post-COVID were present already during the acute phase (25). In contrast, another study (18) reported that transcriptional differences between post-COVID patients and fully recovered individuals became evident but not until six months after infection. Since it is unclear if the molecular changes associated with post-COVID vary over time after the acute infection, a comparison between studies conducted at different follow-up time-points may be difficult. Moreover, prior studies on post-COVID have enrolled patients without distinctions between critical, severe and mild acute COVID infections. An approach to minimize the presence of confounding factors among study participants is to select a homogeneous population of post-COVID patients with similar clinical presentation at the onset of the infection. Furthermore, it is necessary to perform more long-term follow-up studies to better understand the mechanisms underlying the long-lasting symptoms in subsets of patients.

In this study, we aimed to investigate, in PBMCs from a large and homogeneous cohort of patients monitored for a median of 28 months following a mild SARS-CoV-2 infection (1): the presence of transcriptomic changes; (2) the existence of an interferon signature; (3) the persistence of SARS-CoV-2; (4) the differences in post-COVID symptoms and transcriptomic profiles between individuals infected with early variants versus the Omicron variant; and (5) whether capillary lactate levels were ele vated during exertion.

Our study uncovered distinct expression signatures and dysregulated genes enriched in biological processes related to chronic immune response, viral regulation, and mitochondrial dysfunction, whereas no support was found for a persistent infection in PBMCs. Overall, these results indicate that specific transcriptional changes are present in PBMCs of post-COVID patients for years after a mild infection, suggesting multiple mechanisms underlying the post-COVID phenotype that could serve as potential targets for intervention.

## Materials and methods

### Participants

We recruited 60 patients who fulfilled post-COVID criteria according to the WHO classification (1) and 50 sex- and age-matched (± 5 years) control individuals who contracted a SARS-CoV-2 infection during the same time period (+/- 3 months). The control group recovered fully without long-term symptoms or sequelae from the infection. All participants had their COVID infection between February 2020 and April 2022, and all blood samples were collected in April-May 2023.

Inclusion criteria were individuals between 18 and 65 years of age and a previous acute COVID infection that did not require hospitalization. Before inclusion, all patients underwent physical examination, radiological analysis and blood tests to rule out other causes of symptoms. Exclusion criteria were malignant diseases, autoimmune diseases, chronic or ongoing infections, treatment with corticosteroids and/or immunosuppressive drugs, or chronic cardiovascular diseases prior to COVID infection. Information was collected from medical records of all participants, including medical history and clinical parameters at the time of COVID infection. The majority of post-COVID patients was enrolled at the Uppsala post-COVID outpatient clinic and a small proportion was recruited via the National Swedish COVID Association. Matched control individuals were recruited at the primary healthcare center of Östhammar among patients who sought care for symptoms not related to a COVID infection, and among relatives to patients.

Twenty-four post-COVID patients contracted a SARS-Co-V2 infection during the first wave in Sweden (February 2020-August 2020), eight patients had their infection in the second wave (September 2020-February 2021), five patients in the third wave (March 2021-July 2021) and twenty-three in the fourth wave (August 2021-April 2022). Participants who contracted the virus in waves 1–3 were not vaccinated. Participants from the 4th wave, primarily caused by the Omicron variant, were vaccinated with at least 2 shots. All participants had a PCR verified COVID infection except for ten patients and eight controls who got the infection at the very beginning of the pandemic when the access to PCR tests was limited and reserved for severely affected individuals.

The time interval between the COVID infection and sampling was 27.9 ± 8.5 months for all patients (median 28.0 months, range 14–38 months) and 25.8 ± 8.1 months for all controls (median 26.0 months, range 11–39 months). Participating subjects infected in the 4^th^ wave had a time interval between infection and sampling of 19.6 ± 6.0 months for patients (median 16 months, range 14–37 months) and 18.4 ± 5.0 months for controls (median 16 months, range 11–28 months). The corresponding time intervals for participants infected in wave 1, 2 and 3 was 33.1 ± 4.8 months for patients (median 36 months, range 25–38 months) and 32.6 ± 5.2 months for controls (median 32.5 months, range 24–39 months).

The study was approved by the Swedish Ethical Review Authority (2021-06852-0160) and conducted in accordance with the Helsinki declaration. All participants gave written informed consent.

### Clinical assessment

The study protocol was launched in April-Maj 2023 with the collection of clinical data, blood sampling and the application of assessment scales for physical fatigue (Fatigue severity scale, FSS (26), mental fatigue (Mental Fatigue Scale, MFS) (27), depression (Montgomery Asberg Depression Rating Scale, MADRS) (28) and anxiety (Hospital Anxiety and Depression Scale, HAD) (29). The following cut-offs were used after adjustment to the Swedish populations; FSS: score 0-63, cut off ≥ 36; MFS: score 0-42, cut off ≥ 10; MADRS: score 0-54, range 0-12: no depression, 13-19: mild depression, 20–34 moderate depression, and >35: severe depression. HAD depression: score 0-21, cut off ≥ 7. HAD anxiety: score 0-21, cut off ≥ 7. Post-COVID symptom severity was assessed by a score (SSS) based on 17 symptoms on a 10-point scale (0= no symptom, 10 max severity of the symptom), as presented in a previous study (30). Differences in assessment scores between the post-COVID and control groups were measured using a two-tailed two-sample t-test assuming equal variance. Linear correlation was used to compare the proportion of variation between dependent and independent variables expressed as R-squared values.

### Ergometer exertion test

Thirty six patients and thirty one controls performed an exertion session using an ergometer bike (Monark LC4, Vansbro, Sweden) initially set at 25 watt and increased by 25 watt every 5 min, cycling with a revolutions-per-minute (RPM) goal around 50 RPM. Participants were asked to evaluate their effort using the Borg-scale (31) before starting cycling, and then every 5 minutes during the test and 5 min after the test ended. Pulse and oxygen levels in blood was monitored at the same time intervals. Capillary blood was collected from fingertips before the exertion test started (T0, basal value), directly after finishing the test (T1, post-exertion), and then again after approximately 5 minutes of rest (T2, recovery phase, after rest), when the participants’ heart rate had returned to resting frequency.

Capillary tube containing heparin was collected in a hemolyzing agent containing test tube (‘Safe-Lock’ reaction cup 1 000 μL, EKF Diagnostics, Cardiff UK) and analysed by Biosen C*-*Line (EKF Diagnostics) for glucose and lactate (mmol/L). At both T1 and T2, each lactate value was adjusted based on the wattage that each participant achieved during cycling.

### PBMCs isolation and RNA preparation

Eight ml of peripheral whole blood were used for immediate isolation of PBMC using the BD Vacutainer^®^ CPT™ Cell Preparation Tube (BD Biosciences) according to the manufacturer´s instruction (32). Cell pellets were resuspended in RNAprotect^®^ Cell Reagent (Qiagen, cat. no. 76526) and stored at −80 °C. Total RNA was extracted within one week from sampling using the Maxwell^®^ RSC simplyRNA Cells Kit (Promega, ref. AS1390), with the Maxwell^®^ CSC Instrument and Software v3.1.0.177 (Promega, 2800 Woods Hollow Road, Madison, WI 53711-5399, USA).

The quality and quantity of RNA was assessed using Agilent Tape Station 2200 (Agilent, Santa Clara, CA, USA). The RNA concentration varied between 50 to 550 ng/ul, and the RNA integrity number (RIN) were >8.5 in all samples.

### RNA sequencing

Sequencing libraries were prepared from 500ng/μg of polyA selected RNA using the TruSeq stranded mRNA library preparation kit (cat# 20020595, Illumina Inc.). Unique dual indexes (cat# 20022371, Illumina Inc.) were used. The library preparation was performed according to the manufacturers’ protocol (#1000000040498). Sequencing was performed using paired-end 150 bp read length on a NovaSeq X Plus system, 10B flow cell and XLEAP-SBS sequencing chemistry. Samples were analyzed with the nf-core RNA sequencing pipeline release 3.15.1 (nf-co.re/rnaseq) (33). In brief, the pipeline processes raw data from FastQ inputs, aligns the reads, generates counts relative to genes or transcripts and performs extensive quality-control of results.

### Data analysis

Power estimation by read count and dispersion was performed using the R package RnaSeqSampleSize (Release 2.12) (34). The functions est_count_dispersion and est_power_distribution were used to calculate dispersion in our data set to 0.10203, resulting in a power estimation of 0.964 (n=50; rho=2; repNumber=500) at an adjusted p-value (FDR) set to 0.05. We used a random set of 500 genes (repNumber) which gives us a 96.4% probability to find significantly expressed genes between the two groups (n>50) with a minimal fold change of 2 (rho=2) using an adjusted p-value at 0.05 for significance.

Prior to further analysis, genes with zero reads in more than five samples were filtered out. Subsequently, raw counts were pre-processed and used for differential expression analysis by the R package DESeq2 (v1.30.1) (35). Differential expression was performed based on condition (patients vs controls) or by the parameters SSS, MADRS, MFS or Wave within the patient group by subsetting the patients in the analysis. Heatmaps were produced using the R package ComplexHeatmap (v2.6.2) (36) illustrating Z-scores for each gene and clustering was performed on the Euclidean distances between genes and samples. Volcano plot was generated using the R package Enhanced Volcano. Gene Ontology (GO) enrichment for molecular function was performed using the PANTHER Overrepresentation Test (Released 20240807) and the GO Ontology database (released 2024-06-17).

The genes encoding interferons (IFNs) were assigned according to the Interferome V2–0 database (37). Expression values of *IFI27, IFI44L*, *IFIT1*, *ISG15*, *RSAD2*, and *SIGLEC1* were used to calculate an “IFN score” for each individual and according to an established approach to minimize inter-laboratory variability of the IFN signature analysis (38, 39). The comparison of IFN score in the group of post-COVID patients with that of the control group was estimated using an unpaired two-sided t-test.

### Screening for SARS-CoV-2 virus fragments

A screening for SARS-CoV-2 RNA fragments was performed in 53 RNA samples from post-COVID patients alongside with samples from 3 negative controls (consisting of ultra-pure water (Qiagen)) and 1 positive control (consisting of a sample from a former patient infected by omicron BA.1.18). Preparation of sequencing-libraries, including cDNA synthesis, PCR, end-preparation, barcoding and adapter ligation were performed according to the ARTIC protocol (40) with the NEBNext ARTIC SARS-CoV-2 Companion kit reagents #E7660L (New England Biolabs). The ARTIC protocol amplifies numerous 400-base segments representing the entire viral genome and yields hundreds to thousands of copies of each amplicon in positive samples. We applied the VarSkip primer set with 153 primers and the Spike-in primers (version 2024, New England Biolabs) to generate PCR amplicons that cover all known SARS-CoV-2 variants. Three non-template controls were used for normalization. Sequencing and base calling were performed on a GridION sequencer (Oxford Nanopore Technologies), with its integrated software MinKNOW (version 24.06.15). The sequence raw-data were assembled and analysed using Geneious Prime (version 2024.0.7) (41) with an in-house developed bioinformatic workflow (42). The BBDuk plugin (Biomatters Inc.) was used for adapter- and quality trimming. Read mapping against the SARS-CoV-2 reference sequence NC_045512.2 was performed using the Minimap2 plugin (version 2.2.0) (43).

## Results

### Clinical characteristics of patients and controls

Detailed clinical information and responses to rating scales of each individual study participant are presented in **Supplementary Table 1**. The age range of study subjects were between 23 and 64 years with a mean age of 43.6 ± 10.4 years in post-COVID patients (median 44 years, range 23–63 years), and of 44 ± 10.6 years in controls (median 43.5 years, range 24–64 years) (**Table 1**).

**Table 1:** Clinical characteristics and rating scales.

Variable	Patient	Control	P-value
Individuals	n=60	n=50	
Gender (m/f (%f))	13/47 (78%)	11/39 (78%)	0.84
Age	43.6 ± 10.5 (median 44 years, range 23-63)	44 ± 10.6 (median 43.5 years, range 24-64)	0.86
BMI initial	24.5 ± 3.8	24.4 ± 3.6	0.93
BMI current	26.6 ± 5.5	24.4 ± 3.6	0.023
BMI diff	2.2 ± 3.0	0 ± 0	≤0.001
Sick leave	36 of 60	0 of 50	≤0.001
Infection period			
* Wave 1*	24 (40%)	15 (30%)	
* Wave 2*	8 (13%)	6 (12%)	
* Wave 3*	5 (8%)	5 (10%)	
* Wave 4 (Omicron)*	23 (38%)	24 (48%)	
Symptoms duration (months)	27.9 ± 8.5 (median 28.0, range 14-38)	25.8 ± 8.7 (median 26.0, range 11-39)	0.19
Symptoms duration wave 1-3	33.1 ± 4.8 (median 36, range 25-38)	32.6 ± 5.2 (median 32.5, range 24-39)	0.66
Symptoms duration wave 4 (Omicron)	19.6 ± 6.0 (median 16, range 14-37)	18.4 ± 5.0 (median 16, range 11-28)	0.48
Post-COVID Symptom Severity Score			
Total score	61.6 ± 27	9.1 ± 13.1	≤0.001
* Cognitive fatigue*	7.0 ± 2.4	1.4 ± 2	≤0.001
* Physical fatigue*	6.6 ± 2.4	1.1 ± 1.8	≤0.001
* Dyspnea*	4.3 ± 2.9	0.4 ± 1.2	≤0.001
* Heaviness in the chest*	2.9 ± 2.9	0.2 ± 0.9	≤0.001
* Palpitations*	4.2 ± 3.2	0.4 ± 0.9	≤0.001
* Fainting*	2.1 ± 2.4	0.4 ± 1.5	≤0.001
* Dizziness*	3.7 ± 2.6	0.5 ± 1.6	≤0.001
* Diarrhea/abdominal pain*	2.6 ± 2.9	0.3 ± 1.3	≤0.001
* Headache*	4.7 ± 3.3	1.2 ± 2.0	≤0.001
* Myalgia*	4.7 ± 3.3	0.6 ± 1.2	≤0.001
* Fever*	2.6 ± 3.2	0.2 ± 0.9	≤0.001
* Tinnitus*	2.0 ± 2.8	0.3 ± 1.2	≤0.001
* Paresthesia/crawling sensation*	3.0 ± 3.3	0.3 ± 0.8	≤0.001
* Hyposmia/hypogeusia*	1.4 ± 2.8	0.2 ± 0.9	≤0.001
* Anxiety*	3.2 ± 3.1	0.6 ± 1.4	≤0.001
* Depression*	2.9 ± 3.0	0.4 ± 1.1	≤0.001
* Insomnia*	4.2 ± 3.3	0.8 ± 1.7	≤0.001
**MADRS**	15 ± 7.8	4.7 ± 4.8	≤0.001
**HAD anxiety**	6.4 ± 4.0	3.9 ± 3.4	≤0.001
**HAD depression**	6.9 ± 4.2	1.8 ± 2.6	≤0.001
**FSS**	55.7 ± 9.6	21.7 ± 11.1	≤0.001
**MFS**	20.9 ± 6.1	3.3 ± 3.8	≤0.001
Education level			
High education	40	39	0.33
Secondary education	19	11	
Primary education	1	0	
Comorbidity at the time of infection			
Total	27 of 60	23 of 50	0.92
* Allergi/asthma*	11 of 60	10 of 50	
* Hypertension*	1 of 60	2 of 50	
* Depression/anxiety*	6 of 60	8 of 50	
* Hypothyreodism*	2 of 60	1 of 50	
* ADHD*	2 of 60	0 of 50	
* Anorexia*	1 of 60	1 of 50	
* Diabetes*	0 of 60	0 of 50	

Comparison of clinical characteristics and rating scales in post-COVID patients versus controls. Post-COVID Symptom Severity Score (0-170) includes the 17 following symptoms on a 10 point scale (0= no symptom, 10 max severity). MADRS (depression scale 0-54), HAD anxiety (0-21), HAD depression (0-21), FSS (fatigue severity score 0-63), MFS (mental fatigue score 0-42). Data are presented as mean ± SD and compared with Student`s T-test, and as median with range where indicated.

Bold values show independent rating scales.

The majority of patients (78%) were females. Patients and controls had a comparable socioeconomic status as well as a similar educational level (p-value = 0.33), that was high in 72% of all participants. Both groups had a similar BMI at the time of COVID infection (p-value = 0.93). The patient group, but not the control group, had gained weight (2.2 BMI) at the time of blood sampling, likely because of inactivity. Comorbidity rate at the time of infection was similar for patients and controls (**Table 1**). Most patients were in good health and physically active before infection. At the time of blood sampling and post-COVID assessment, sixty percent of patients were on sick leave and a majority reported poor health and reduced quality of live at the EQ-5D-5L scale. All rating scales showed significant differences when comparing the group of patients with the control group. Patients reported higher levels of both physical fatigue (FSS 55.7 ± 9.6) and mental fatigue (MFS 20.9 ± 6.1), when compared to healthy controls (p-value <0.001). Depression rating using MADRS indicated a mild depression among patients (15.9 ± 7.8), whereas HAD rating for depression in the patient group were just below the cut off (6.9 ± 4.2). Patients did not meet the criteria for anxiety (HAD anxiety 6.4 ± 4). However, the levels of depressive symptoms and anxiety were significantly higher (p-value <0.001) among patients when compared to the control group (**Table 1**). The post-COVID symptom severity score (SSS; scale 0-170) was 61.6 ± 27 for patients and 9.1 ± 13.1 for controls (p-value <0.001). The most disabling symptoms reported among patients (scale between 0-10) were cognitive fatigue (7.0 ± 2.4), physical fatigue (6.6 ± 2.4) and dyspnea (4.3 ± 2.9) (**Table 1**). There was no correlation between the post-COVID symptom severity score and patients age (R² = 0,0238) (**Supplementary Figure 1A**). A comparison between patients who were infected with the earlier COVID variants (wave 1-3) and patients who contracted the Omicron variant (fourth wave) revealed no significant differences in self-reported post-COVID symptoms (p-value >0.05) (**Supplementary Figure 1B**).

### Ergometer exertion test results

Thirty-six patients and thirty-one controls performed an ergometer exertion test. In the patient group, three subjects cycled at 50 watts, seven at 75 watts, twenty-one at 100 watts, four at 125 watts, and only one at 150 watts. Dyspnea and physical exhaustion were the primary reasons for test interruption. In the control group, one subject cycled at 100 watts, two at 125 watts, twenty-four at 150 watts, and four at 175 watts. No participant experienced desaturation, with oxygen saturation levels remaining at least 94%. The mean resting heart rate was 76.4 beats per minute in the patient group and 69.6 beats per minute in the control group. Of note, 10 patients were on beta-blockers or Ivabradine. Analysis of lactate levels before the cycle ergometer test (T0, basal level) revealed significantly higher levels of lactate in capillary blood in the patient group compared to controls (p-value = 0.011). At T1, immediately after the end of exertion, patients exhibited slightly higher lactate levels than controls but the difference was not statistically significant (p-value= 0.14). At T2, after five minutes of rest, patients again showed significantly higher lactate levels compared to controls, when adjusted for workload (p-value < 0.01) (**Supplementary Figure 2**).

### Differentially expressed genes in patients vs. controls

**Figure 1:**
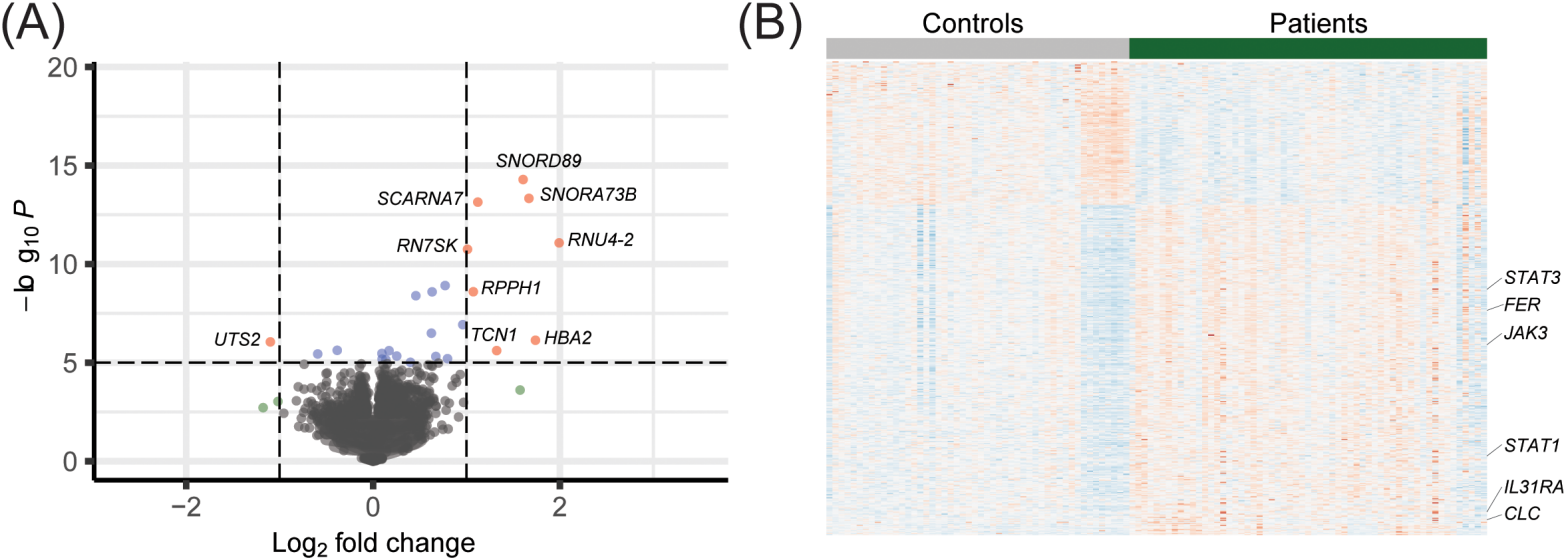
Gene expression profiles of post-COVID patients compared to controls. **(A)** Volcano plot illustrating differentially expressed genes in the patients and control individuals. **(B)** Heat-map showing differentially expressed genes in patients and control individuals. Six upregulated genes that are enriched in the GO term JAK/STAT signaling pathway are highlighted.

The RNA-sequencing resulted in an average of 48M reads (span 28 – 75M). One sample was removed due to low coverage (11M reads). The correlation between samples was > 0.96 (**Supplementary Figure 3**). Analysis of differentially expressed genes between the patients and control groups identified a total of 463 transcripts (adjusted p-value <0.05) of which 324 transcripts had a higher expression and 139 transcripts had a lower expression in the patient group (**Figure 1**; **Supplementary Table 2**). The 13 top dysregulated transcripts are presented in **Table 2**.

**Table 2:** Most differentially expressed genes in patients versus controls.

Gene	Transcript	log2FC	padj
Upregulated genes			
*RNU4-2*	ENSG00000202538.1	2.0	3.64E-08
*HBA2*	ENSG00000188536.12	1.7	0.0011
*SNORA73B*	ENSG00000200087.1	1.7	4.03E-10
*SNORD89*	ENSG00000212283.1	1.6	8.94E-11
*LTF*	ENSG00000012223.12	1.6	0.027
*TCN1*	ENSG00000134827.7	1.3	0.0027
*SCARNA7*	ENSG00000238741.1	1.1	4.16E-10
*RPPH1*	ENSG00000277209.1	1.1	5.56E-06
*RN7SK*	ENSG00000202198.1	1.0	6.16E-08
*ANXA3*	ENSG00000138772.12	1.0	0.044
*HIST1H1E*	ENSG00000168298.5	1.0	0.000251
Downregulated genes			
*UTS2*	ENSG00000049247.13	-1.1	0.0012
*EGR1*	ENSG00000120738.7	-1.0	0.042

Differentially expressed genes and transcripts (adjusted p-value < 0.05) with a log2 fold change (log2FC) equal to or higher than 1 (upregulated genes) or lower than -1 (downregulated genes). Benjamini and Hochberg adjusted p-values (padj) from DESeq2.

Among the most upregulated transcripts in post-COVID patients we identified a group of non-coding RNAs, such as small nuclear RNAs (snRNAs), small nucleolar RNAs (snoRNAs) and long non-coding RNA (lncRNAs). The single most upregulated snRNA was *RNU4–2 U4* (log2 fold change 2.0), a critical component of the major spliceosome. Furthermore, several small nucleolar RNAs involved in RNA processing, such as *SNORA73B*, *SNORD89* and *scaRNA7*, showed a marked increase in expression levels (log2 fold change 1.7, 1.6, and 1.1, respectively). We also identified overexpression of the long non-coding RNA *RPPH1* (Ribonuclease P RNA Component H1), important for the cleavage of tRNA precursor molecules and the formation of mature 5′termini of tRNA sequences, as well as for the expression of pro-inflammatory cytokines and cell proliferation (44) (log2 fold change 1.0).

The most upregulated protein-coding genes were *HBA2*, *TCN1*, *LTF*, *ANXA3* and the histone H1 family member *HIST1H1E* (**Table 2**). The *HBA2* gene, encoding hemoglobin subunit alpha 2 showed a log2 fold change of 1.7, suggesting potential alteration in oxygen transport. Furthermore, we found an upregulation of the genes *LTF* (log2 fold change 1.6), *ANXA3* (log2 fold change 1.0), and *TCN1* (log2 fold change 1.3) that encode for the proteins Lactoferrin, Annexin3, and Transcobalamin1, respectively, all of which are associated with neutrophil degranulation and vesicular transport.

### Enrichment in GO terms of differentially expressed genes

We then sought to investigate the predicted effects of differentially expressed genes on known functional pathways. Enrichment analysis of upregulated genes in post-COVID patients revealed that the top GO terms for biological functions were Negative regulation of protein autoubiquitination (2 genes; fold enrichment 94.03; adjusted p-value 1.12x10-4), Interleukin-9 signaling pathway (3 genes; fold enrichment 40.3; adjusted p-value 1.39x10-2) and JAK-STAT signaling pathway (6 genes; fold enrichment 11.1; adjusted p-value 7.11x10-3). Additional biological processes enriched for overexpressed genes were Negative regulation of viral processes (7 genes; fold enrichment 6.9; adjusted p-value 2.2x10-2) and Defense response to virus (11 genes; fold enrichment 3.8; adjusted p-value 1.67x10-4) (**Table 3**). Conversely, enrichment analysis of downregulated genes in post-COVID patients revealed five top GO terms for processes of DNA repair and replication, followed by the terms Aerobic electron transport chain, Mitochondrial ATP synthesis and Oxidative phosphorylation (**Table 3**).The complete list of enriched GO terms of biological process has presented in **Supplementary Table 3**.

**Table 3:** Enrichment analysis of differentially expressed genes.

GO biological process	Genes	Fold enrichment	padj
Upregulated genes			
negative regulation of protein autoubiquitination (GO:1905524)	2	94	0.030
interleukin-9-mediated signaling pathway (GO:0038113)	3	40.3	0.014
cellular response to interleukin-9 (GO:0071355)	3	40.3	0.014
response to interleukin-9 (GO:0071104)	3	35.3	0.020
receptor signaling pathway via JAK-STAT (GO:0007259)	6	11.1	0.007
receptor signaling pathway via STAT (GO:0097696)	6	10.3	0.011
negative regulation of viral process (GO:0048525)	7	6.9	0.022
regulation of RNA splicing (GO:0043484)	9	4.5	0.045
regulation of mRNA metabolic process (GO:1903311)	14	4.3	0.003
chromatin remodeling (GO:0006338)	28	4.2	0.000
defense response to virus (GO:0051607)	11	3.8	0.041
positive regulation of innate immune response (GO:0045089)	11	3.7	0.048
chromatin organization (GO:0006325)	29	3.6	0.000
protein-DNA complex organization (GO:0071824)	30	3.3	0.000
positive regulation of defense response (GO:0031349)	14	3.2	0.036
Downregulated genes			
double-strand break repair via break-induced replication (GO:0000727)	4	54.5	0.003
regulation of DNA-templated DNA replication initiation (GO:0030174)	4	43.6	0.003
mitotic DNA replication (GO:1902969)	3	35	0.030
DNA unwinding involved in DNA replication (GO:0006268)	4	31.1	0.006
DNA replication initiation (GO:0006270)	4	23.4	0.014
maturation of LSU-rRNA (GO:0000470)	4	23.4	0.013
aerobic electron transport chain (GO:0019646)	6	11.1	0.011
DNA duplex unwinding (GO:0032508)	5	10.8	0.038
mitochondrial ATP synthesis coupled electron transport (GO:0042775)	6	10.5	0.014
ATP synthesis coupled electron transport (GO:0042773)	6	10.5	0.014
cytoplasmic translation (GO:0002181)	8	10.2	0.003
DNA geometric change (GO:0032392)	5	10	0.047
electron transport chain (GO:0022900)	9	8.8	0.003
respiratory electron transport chain (GO:0022904)	6	8.5	0.031
oxidative phosphorylation (GO:0006119)	6	8.2	0.036

Enrichment analysis of differentially expressed genes, using PANTHER Overrepresentation Test and the GO Ontology database. The column Genes shows the number of differentially expressed genes belonging to each GO category. Fold enrichment is based on the number of observed differentially expressed genes in each category compared to the expected adjusted p-value (padj) < 0.05 for the enrichment. The top upregulated pathways in post-COVID comprise negative regulation of ubiquitination, IL-9 signaling, JAK/STAT signaling and defense mot virus. The top downregulated pathways involve DNA repair and replication, and mitochondrial ATP synthesis.

### JAK/STAT signaling

**Figure 2:**
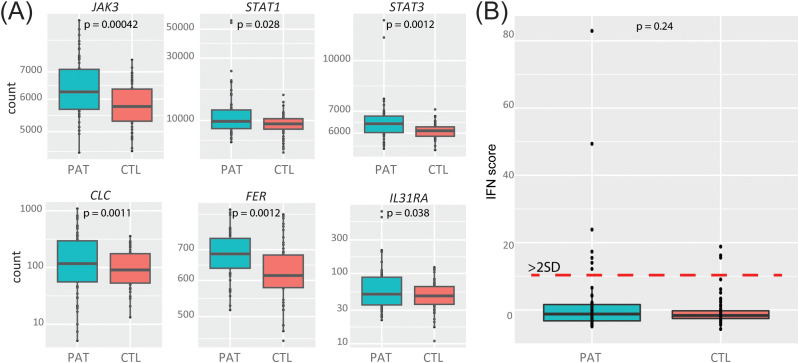
Differential expression of JAK/STAT genes and interferon signature in post-COVID patients and controls. **(A)** Differentially expressed genes belonging to the JAK-STAT pathway. **(B)** Interferon (IFN) score was similar in the two groups (p=0.24).

The enrichment of up-regulated genes in the post-COVID group that belong to the JAK-STAT signaling pathway, previously proposed to play a role for severe manifestations in SARS-CoV-2 infections, made us investigate what specific genes were dysregulated. The six upregulated and enriched genes in JAK-STAT signaling comprised *JAK3, STAT1, STAT3, CLC, FER* and *IL31A* (log2 fold change: 0,1-0,8; adjusted p-value 0,028-0,048; **Figure 2A**).

### Interferon signature

When we calculated the IFN score, using the expression values of six INF genes, as described in Material and Methods, we did not detect any differences when comparing the post-COVID patients with the control group (IFN-score: controls = 0.0 ± 51; patients = 2.4 ± 13.6; p-value = 0.24) (**Figure 2B**).

### Correlation between transcriptome analysis and clinical parameters within the group of patients

We then searched for differentially expressed genes within the post-COVID group by using the self-reported post-COVID symptom severity score (SSS), the scores for mental fatigue (MFS), physical fatigue (FSS), and depression (MADRS) as variables. No differentially expressed genes could be identified as associated with sub-groups of post-COVID patients based on their self-reported SSS, FSS, MADRS or MFS (adjusted p-value < 0.05 and log2 fold change > 0.2 or < -0.2). However, the self-reported intensity of “dyspnea”, a clinical parameter included in the SSS assessment, resulted correlated with the expression of 13 genes (*ALAS2, SLC4A1, LTF, HBA2, HBA1, HBB, PHOSPHO1, HBD, CA1, ANXA3, SELENBP1, DEFA3, CAMP*). Notably, three of these genes ae hemoglobin genes of importance for oxygen transport (**Supplementary Table 4**).

Furthermore, to assess whether patients infected during the first three waves (unvaccinated and exposed to more aggressive virus variants) exhibited a distinct transcriptomic profile from those infected in the fourth wave (Omicron variant), we compared the transcriptome profiles between the two groups. Our analysis did not reveal any differentially expressed transcripts (adjusted p-value < 0.05 and log2 fold change > 0.2 or < -0.2).

### SARS-CoV-2 gene fragment screening in patients

**Figure 3:**
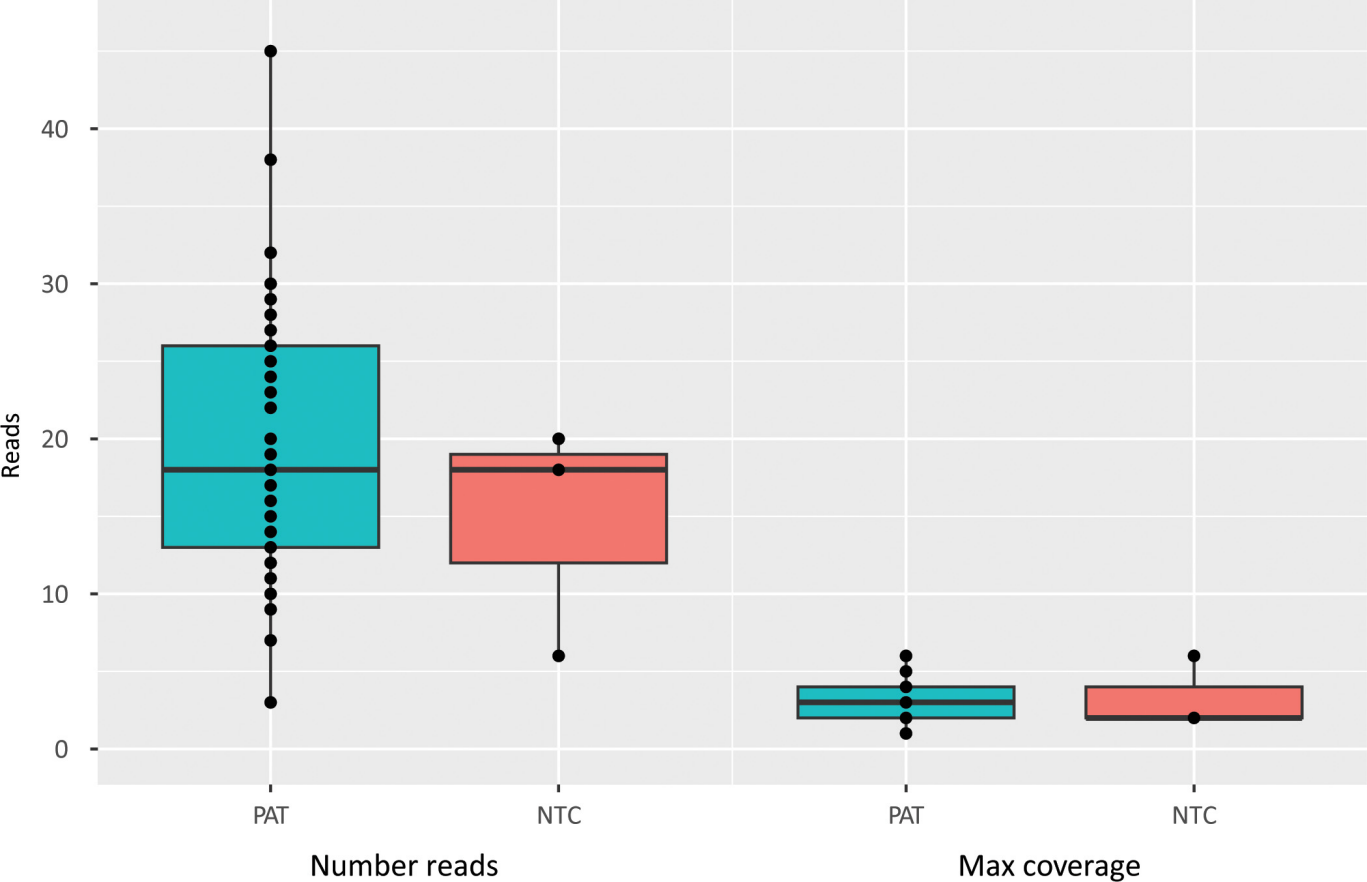
Screening for SARS-CoV-2 virus RNA fragments. Number of sequencing reads and sequencing coverage of SARS-CoV-2 virus RNA in patient samples (patients) and in samples from negative control individuals (NTC, non template control).

It has been suggested that persistent infection may contribute to post-COVID. To investigate the presence of remaining SARS-CoV-2 in post-COVID patients, we analyzed PBMC-RNA from a positive control, three negative control samples and 53 patients from whom we had remaining RNA. From the positive control we obtained 87764 sequence reads with a maximum coverage of 4926. In comparison, the three negative controls yielded between 6 and 20 reads per sample, with a maximum coverage ranging from 2 to 6. The analysis on PBMC-RNA from 53 post-COVID patients revealed between 3 and 45 reads, with a maximum coverage of 1 to 6 (**Figure 3**). The results from the 53 patient samples were thus comparable with results from the three negative control samples without SARS-CoV-2 gene fragments.

## Discussion

The mechanisms and pathophysiology behind post-COVID are still elusive. In this report we present genome-wide transcriptomic changes associated with post-COVID at a median follow-up of 28 months following a mild acute COVID infection. The study is powered by a large and homogeneous cohort, which, together with the very long follow-up time, brings additional important information on persistent molecular changes associated to the post-COVID phenotype.

The transcriptional analysis of PBMCs uncovered 463 differentially expressed genes (adjusted p-value < 0.05) of which 324 were upregulated and 129 were downregulated. The most pronounced enrichment of up-regulated genes was identified in the GO-term “Negative regulation of autoubiquitination”. The GO category “Negative regulation of protein autoubiquitination” contains only two genes, namely *TAF1* och *MARCHF7*, both of which were up-regulated. Ubiquitination of proteins for the recognition and degradation by the proteasome is a central mechanism in the defense against virus, included SARS-CoV-2 (45). Many viruses encode for proteins that can subvert or manipulate the host’s ubiquitination machinery to evade the host-immune response and to facilitate viral replication (46). Impaired ubiquitination may reduce the possibility for clearance and inactivation of virus whereas elevated ubiquitination has been shown have a protective role against severe SARS-CoV-2 infection (47). Furthermore, several autoimmune diseases are associated with impaired ubiquitination (48–50), which can be triggered by viral infections such as EBV (51). It is therefore tempting to hypothesize that SARS-CoV-2 infection, through dysregulation of ubiquitination and impaired proteasome degradation, may lead to an activation of innate immune cells, which, together with other mechanisms, contribute to chronic inflammation and possibly autoimmunity. It has been reported that SARS-CoV-2 infection is linked to a substantially increased risk for the development of a broad spectrum of new-onset autoimmune diseases, including rheumatoid arthritis, systemic lupus erythematosus, inflammatory bowel disease and type 1 diabetes (52, 53). It is important to note, however, that in our study we excluded post-COVID patients who developed ANA positivity or autoimmune diseases following the infection.

We also observed strong up-regulation of the GO terms IL-9-mediated signaling and Response to IL-9 in our post-COVID group. Interleukin 9 causes airway inflammation in both infectious and allergic diseases (54). Moreover, IL-9 has been shown to aggravate infection and exacerbate associated airway inflammation in a mouse model of SARS-CoV-2 (55). In our study, the number of participants with asthma and allergy was the same in patients and control groups. Therefore, the increase in IL-9 cannot be due to previous hypersensitivity, but more likely a residual effect of SARS-CoV-2 infection.

Furthermore, our group of post-COVID patients showed a marked upregulation of the JAK-STAT signaling pathway. In the acute phase of acute SARS*-*CoV*-*2 infection, JAK-STAT signaling amplifies the pathogenic effect of virus, inducing the release of inflammatory cytokines such as tumour necrosis factor alpha (TNF-α) implicated in cytokine storm and ARDS (56).

In addition, JAK activation contributes to vascular and thrombotic manifestations in the acute phase of infection (57), and a significant correlation has been observed between JAK-STAT signaling pathway and angiotensin-converting enzyme 2 (ACE2) (58). In line with these observations, JAK inhibitors have successfully been used for the treatment of severe pneumonia in COVID-19 (59, 60). The activation of genes belonging to the JAK/STAT pathway has previously been observed in the early phase of the post-COVID condition (15). Our study adds to this finding by demonstrating a persistent activation of JAK/STAT signaling in post-COVID patients for a median of 28 months after infection. JAK/STAT signaling is activated in many physiological processes, particularly in response to cytokine stimulation and growth regulation. However, dysregulation of JAK/STAT signaling may also be implicated in the pathogenesis of inflammatory and autoimmune diseases (61), and JAK inhibitors are successfully used in the treatment of rheumatoid arthritis, inflammatory bowel disease, alopecia areata, multiple sclerosis, Sjögren’s syndrome, Behçet’s syndrome and vasculitis (62). The hypothesis that immunomodulatory treatments may be effective in post-COVID conditions is currently tested in ongoing clinical trials. To our knowledge, at least one randomized clinical trial is currently investigating the effects of the JAK inhibitor Baricitinib on neurological and cardiovascular symptoms associated with post-COVID (REVERSE-LC, ClinicalTrials.gov Identifier: NCT06631287. https://clinicaltrials.gov/study/NCT06631287). Notably, our study did not uncover any differentially expressed IFN genes when comparing the patient and control groups. Interferons are important in the early phases of viral infection and they have been shown to protect airway epithelial cells from SARS*-*CoV*-*2 (63). Blocking IFN signaling is an early mechanism of SARS-CoV-2 to delay innate immune system recognition. In a study in post-COVID patients, the expression of IFN genes declined with time up to eight months after infection (19). A time-dependent decline in the expression of IFN genes is consistent with our observations, showing no remaining interferon activation in the post-COVID group of patients.

The most upregulated specific genes in our study belong to a group of non-coding RNAs. Non-coding RNAs, such as miRNA, may influence the host cells’ innate immune response to viral infections (64). In particular, we found a marked upregulation of the small nucleolar RNAs *SNORA73B*, *SNORD89* and *scaRNA7*. Beside their well-defined roles in ribosomal RNA processing, emerging evidences have shown snoRNAs involvement in the replication of RNA viruses (65). For example, the expression of snoRNAs has been reported significantly altered during influenza A infection (66), moreover *SNORD44*, *SNORD76*, *SNORD78* and *SNORD83* have been described upregulated after viral infection by Chikungunya fever virus (67). A variety of cellular functions are likely affected by the altered expression patterns of ncRNAs, making it difficult to predict the consequences of their increased expression in post-COVID patients. The reason why snoRNAs are overexpressed such a long time after the acute infection in our group of patients remains unclear.

Among the most strongly upregulated protein-coding genes, we identified *TCN1* (encoding Transcobalamin 1), *LTF* (encoding Lactotransferrin) and *ANXA3* (encoding Annexin A3). Interestingly, the three proteins have previously been described as overexpressed in saliva during the acute phase of SARS-CoV-2 infection (68). Moreover, the Annexin family of proteins has been reported to be involved in attachment, internalization, and release of several viruses including hepatitis C virus (HCV) (69) and SARS-CoV-2 (70). However, the reason for the specific overexpression of these proteins after a median follow-up of 28 months following the infection remains obscure.

The enrichment analysis of down-regulated genes in our PASC-cohort uncovered five top GO terms for biological processes of DNA replication and repair. The finding is consistent with an increased DNA damage and cellular senescence following the SARS-CoV-2 infection (71). Furthermore, we identified a strong enrichment of down-regulated genes in processes localized to the mitochondria.

The specific GO terms identified were Aerobic electron transport chain, Mitochondrial ATP synthesis coupled to electron transport chain and Oxidative phosphorylation. Our results are in line with those reported by Ryan et al. (18), who also reported down-regulated oxidative phosphorylation in post-COVID. Notably, the upregulation of *HBA2* in our post-COVID patients may suggest a potential link to altered oxygen transport. Additionally, the elevated blood lactate levels observed both at rest and post-recovery following exertion suggest metabolic dysfunction and increased anaerobic activity in skeletal muscles. Furthermore, the self-reported intensity of “dyspnea” within the group of post-COVID patients correlates with the expression levels of thirteen genes, including three hemoglobin genes. Collectively, these observations reinforce the hypothesis of impaired oxygen delivery and mitochondrial dysfunction. For clinical parameters other than dyspnea, such as the self-reported post-COVID severity symptoms and the mental or physical fatigue, we did not found any correlation to transcriptomic changes. This lack of association may be due to the limited size of our cohort and the characteristic fluctuation of symptom intensity over time in post-COVID condition. Additionally, it is possible that mechanisms beyond the transcriptomic changes identified in our study contribute to the disease’s pathophysiology and influence the dynamic presentation and variability of symptoms.

This study is, to our knowledge, the longest follow-up study of transcriptomic changes associated with post-COVID. The data highlight several molecular alterations and perturbed processes that can be linked to the clinical manifestations in our cohort of patients. However, a remaining fundamental question is what mechanisms underlie the sustained transcriptional changes. Viral reservoirs that maintain the inflammatory response have been suggested as one possible explanation for post-COVID (11, 12). In our study we could not detect any traces of virus in PBMC. On the other hand, we cannot exclude a viral reservoir in other tissues than PBMC as a trigger for the persistent immune responses in our patients. Another possible explanation is that, despite the absence of the virus, mechanisms activated during the acute phase of the infection are maintained through a loop of autoreactivity. Interestingly, patients who contracted the infection in the first three waves of the pandemic (33 months follow-up) showed similar transcriptomic changes as those who contracted the omicron variant in wave four (19,6 months follow-up). This suggests that the post-COVID-associated transcriptional changes in PBMCs are stable over time.

A major strength of our study is the long follow-up time of post-COVID symptoms extended over more than two years in patients with the pre-omicron variant, accompanied by a detailed clinical assessment and a genome-wide analysis of transcriptional changes. Another strength is the relatively large number of study subjects and the homogeneity of the patient and control groups regarding ethnicity, socioeconomic back-ground, severity and time of acute infection.

A potential weakness is the unselected population of PBMCs used for RNA sequencing that does not allow for the assignment of specific transcriptional changes to sub-populations of cells. We did not perform flow cytometry analysis of PBMCs nor any immunophenotyping of different cell populations. Another limitation is that tissues other than PBMCs, were not be investigated for the presence of virus.

In conclusion, the combined data in our study support the hypothesis that post-COVID is a chronic inflammatory disease and highlights JAK/STAT signaling as a potential therapeutic target. Further studies are now required to clarify the mechanisms behind the dysregulated genes and processes involved in the pathophysiology of post-COVID, which may have implications for future pharmacological interventions.

## Data Availability

The data presented in the study is deposited in the SciLifeLab FigShare data repository (https://doi.org/10.17044/scilifelab.28832492).
