# Supplementary material for: Comprehensive transcriptome assessment in PBMCs of post-COVID patients at a median follow-up of 28 months after a mild COVID infection reveals upregulation of JAK/STAT signaling and a prolonged immune response
Source: Front Immunol. 2025 May 30;16:1589589. doi: 10.3389/fimmu.2025.1589589 (PMC12162955; doi:10.3389/fimmu.2025.1589589)
Supplement: Supplementary file 5 [file DataSheet1.pdf]

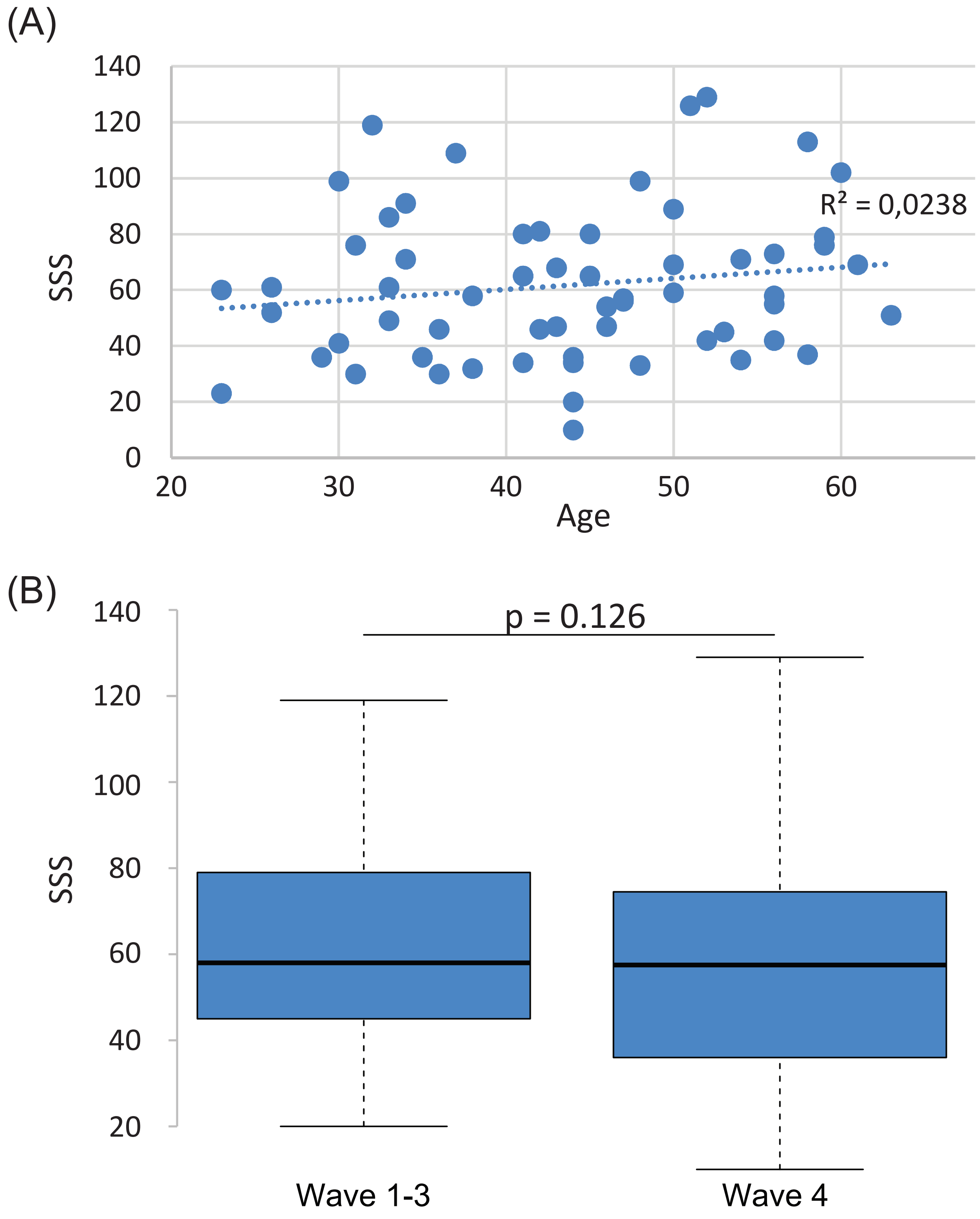

Figure S1. A) Post-COVID symptom severity score showed no correlation with age ($R^2 = 0{,}0238$). B) Post-COVID symptom severity score was not different between patients who contracted the omicron variant (fourth wave) and patients infected by earlier COVID variants (wave 1-3) (p=0.126). Data are presented in boxplots with median and compared with Student`s T-test.
