# Supplementary material for: Comprehensive transcriptome assessment in PBMCs of post-COVID patients at a median follow-up of 28 months after a mild COVID infection reveals upregulation of JAK/STAT signaling and a prolonged immune response
Source: Front Immunol. 2025 May 30;16:1589589. doi: 10.3389/fimmu.2025.1589589 (PMC12162955; doi:10.3389/fimmu.2025.1589589)
Supplement: Supplementary file 6 [file DataSheet2.pdf]

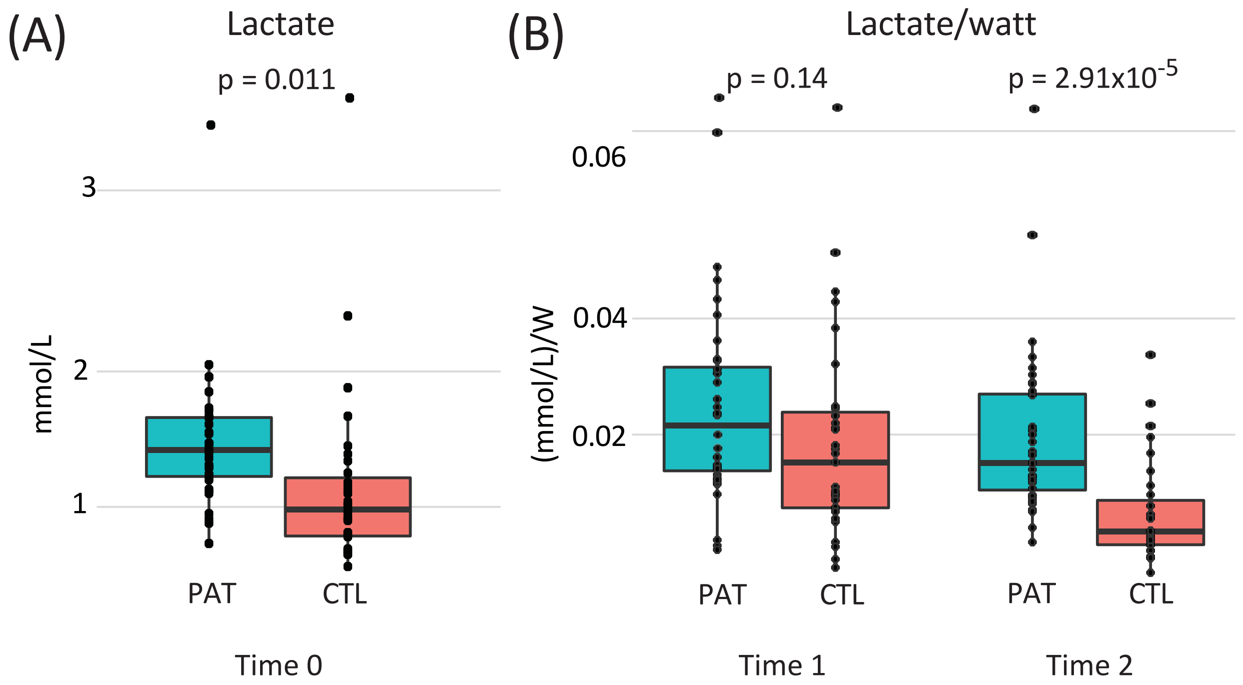

Figure S2. Ergometer exertion test results in post-COVID patients (PAT) and controls (CTL). A) Time 0 is the basal value before the test. Lactate in capillary blood was higher in patients (blue) than controls (red) ($p=0.011$). B) Time 1 is immediately post exertion. Lactate/watt was not different between patients (blue) and controls (red). Time 2 is 5 minutes after rest. The patients had a higher lactate/watt than controls ($p <0.01$). Data was compared using Student`s t-test (p-value). Patients (PAT; n = 36), controls (CTL; n = 31)
