# Supplementary material for: Comprehensive transcriptome assessment in PBMCs of post-COVID patients at a median follow-up of 28 months after a mild COVID infection reveals upregulation of JAK/STAT signaling and a prolonged immune response
Source: Front Immunol. 2025 May 30;16:1589589. doi: 10.3389/fimmu.2025.1589589 (PMC12162955; doi:10.3389/fimmu.2025.1589589)
Supplement: Supplementary file 7 [file DataSheet3.pdf]

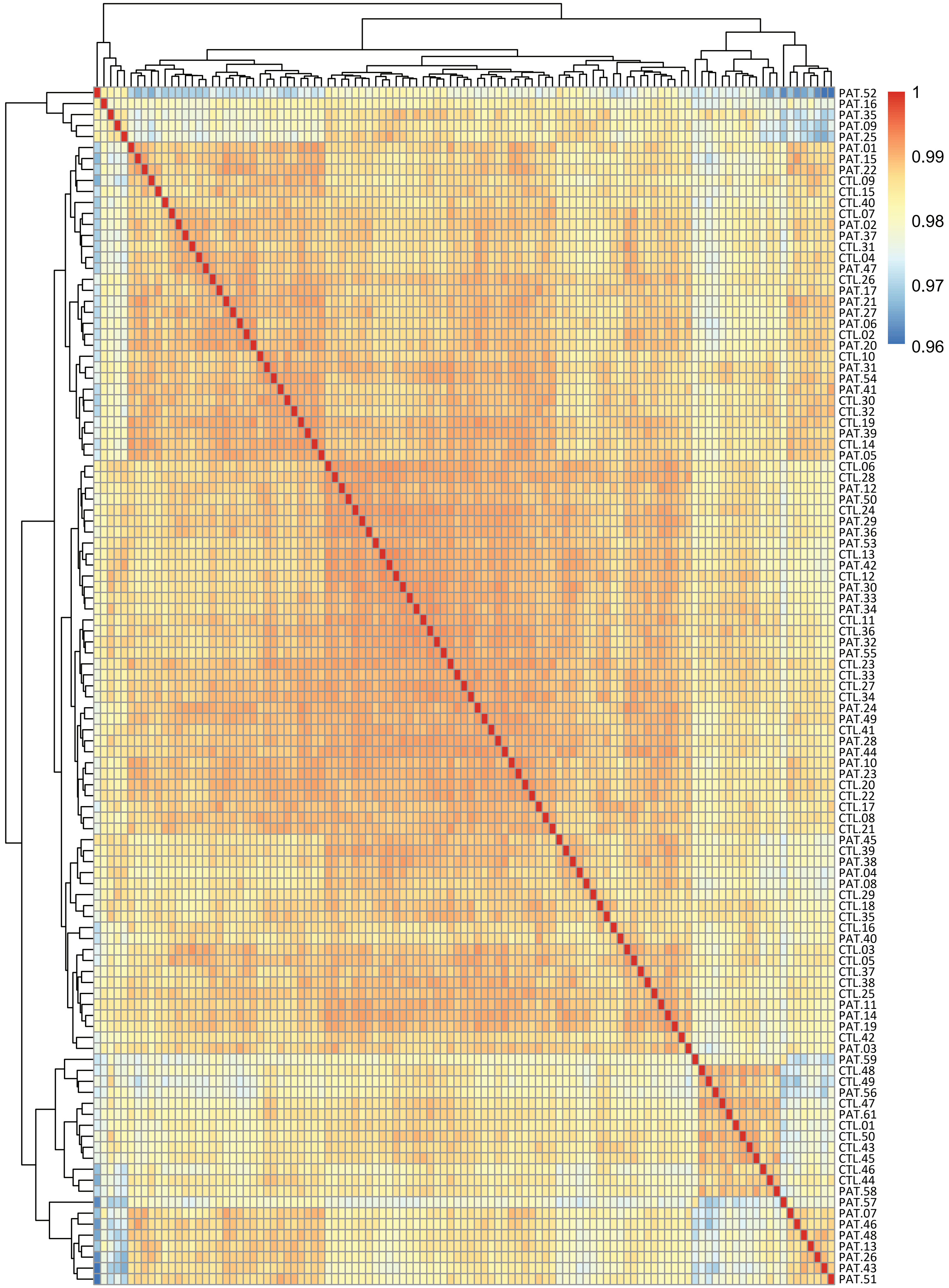

Figure S3. Heatmap over sample correlation. Sequencing of resulted in an average of 48M reads (28 – 75M). One sample was removed due to low coverage (11M reads). The overall correlation between samples was > 0.96. Samples are annotated according to group (patient - PAT, Control - CTL) and number.
